# Minimally Invasive Coronary Artery Bypass Grafting (MICS-CABG) Versus Conventional Off-Pump Coronary Artery Bypass Grafting (OPCAB): A Single-Center Analysis of Early Outcomes and Institutional Experience

**DOI:** 10.7759/cureus.116351

**Published:** 2026-09-16

**Authors:** Muhammad Yasir Khan

**Affiliations:** 1 Cardiac Surgery, Mukhtar A Sheikh Hospital, Multan, PAK; 2 Cardiac Surgery, Sardar Fateh Muhammad Khan Buzdar Institute of Cardiology, DG Khan, PAK

**Keywords:** learning curve, minimally invasive coronary artery bypass, minimally invasive coronary artery bypass off-pump coronary artery bypass learning curve thoracotomy postoperative hemorrhage, minimally invasive surgery, off-pump coronary artery bypass, postoperative hemorrhage, thoracotomy

## Abstract

Background

Minimally invasive coronary artery bypass grafting (MICS-CABG) through an anterolateral mini-thoracotomy is an alternative to conventional coronary artery bypass grafting through median sternotomy. However, evidence regarding its early clinical outcomes and institutional learning phase remains limited in developing healthcare systems.

Objectives

To compare the baseline clinical characteristics, intraoperative parameters, and early postoperative outcomes of patients undergoing MICS-CABG with those undergoing conventional off-pump coronary artery bypass grafting (OPCAB).

Methods

This retrospective cohort study included consecutive patients with multivessel coronary artery disease (CAD) who underwent surgical coronary revascularization at our cardiovascular institute. Patients undergoing non-CABG cardiac procedures or those with incomplete clinical records were excluded. Data were collected on demographic characteristics, cardiovascular comorbidities, operative parameters, duration of postoperative mechanical ventilation, chest tube drainage, and early postoperative complications.

Results

A total of 80 patients were included, comprising 40 patients who underwent MICS-CABG and 40 who underwent conventional OPCAB. Both groups had a high prevalence of cardiovascular risk factors, including type 2 diabetes mellitus and hypertension. The mean duration of postoperative mechanical ventilation was 8.80 ± 3.97 hours in the MICS-CABG group compared with 4.22 ± 2.64 hours in the OPCAB group. Mean postoperative chest tube drainage was 887.0 ± 611.8 mL in the MICS-CABG group and 328.9 ± 116.1 mL in the OPCAB group. No perioperative mortality occurred in either group, and no conversion from MICS-CABG to median sternotomy was observed.

Conclusion

MICS-CABG was technically feasible in this early institutional experience, with no perioperative mortality or conversion to sternotomy. However, patients undergoing MICS-CABG had longer postoperative mechanical ventilation and greater chest tube drainage than those undergoing conventional OPCAB. These findings support the feasibility of MICS-CABG while highlighting the need for continued evaluation of outcomes as institutional experience increases.

## Introduction

Conventional coronary artery bypass grafting (CABG) through a full median sternotomy remains an established surgical approach for patients with complex multivessel coronary artery disease (CAD). Although this approach provides excellent surgical exposure and facilitates complete myocardial revascularization, median sternotomy is associated with potential disadvantages, including postoperative pain, prolonged recovery, impaired sternal stability, and the risk of sternal wound complications [1,2].

In an effort to reduce surgical trauma while maintaining effective myocardial revascularization, minimally invasive coronary artery bypass grafting (MICS-CABG) through a left anterolateral mini-thoracotomy has emerged as an alternative to conventional sternotomy-based CABG [2,3]. By avoiding median sternotomy, MICS-CABG may preserve chest wall integrity and potentially facilitate earlier postoperative recovery, improved mobility, and shorter hospitalization [1,3]. Advances in surgical instrumentation, visualization, coronary stabilization, and peripheral cannulation strategies have contributed to the expanding application of minimally invasive approaches to coronary revascularization.

Despite these potential benefits, establishing a MICS-CABG program presents important technical challenges. The procedure requires coronary exposure and stabilization through a limited surgical field, modified techniques for conduit harvesting and multivessel grafting, and close coordination among the surgical, anesthetic, perfusion, and perioperative teams [1,4]. These technical and organizational demands are particularly relevant during the early implementation of a MICS-CABG program. Evaluation of outcomes during this initial institutional experience is therefore important for assessing procedural feasibility, identifying areas requiring technical refinement, and informing the safe development of minimally invasive coronary revascularization programs.

Data describing the early institutional experience with MICS-CABG in developing healthcare systems remain relatively limited. Therefore, this study aimed to describe our early institutional experience with MICS-CABG and compare baseline clinical characteristics, intraoperative parameters, and early postoperative outcomes with those of patients undergoing conventional off-pump coronary artery bypass grafting (OPCAB) at our institution.

## Materials and methods

This retrospective, single-center comparative cohort study included patients with CAD who underwent surgical myocardial revascularization at Mukhtar A Sheikh Hospital, Multan, Pakistan, between 2023 and 2025. The institutional database and medical records were retrospectively reviewed to identify eligible patients who underwent either MICS-CABG or conventional OPCAB. The study was approved by the Institutional Review Board/Ethics Committee of Mukhtar A Sheikh Hospital on August 8, 2026 (approval number: MASH/2026/27). The choice of surgical approach was made by the operating surgeon based on preoperative clinical assessment, coronary anatomy, technical suitability for minimally invasive access, anticipated ability to achieve adequate revascularization, and patient-specific operative considerations. MICS-CABG was preferentially considered in patients in whom the coronary anatomy and clinical condition were considered suitable for minimally invasive multivessel revascularization, whereas conventional OPCAB was performed in patients managed through the established sternotomy-based revascularization approach. Treatment allocation was not randomized, and no propensity-score matching was performed. Consequently, the possibility of selection bias inherent to the retrospective non-randomized design was recognized.

Patients were selected according to predefined eligibility criteria. The inclusion and exclusion criteria are summarized in Table 1.

**Table 1:** Inclusion and Exclusion Criteria CAD: coronary artery disease; MICS-CABG: minimally invasive coronary artery bypass grafting; OPCAB: off-pump coronary artery bypass grafting.

Inclusion criteria	Exclusion criteria
Age ≥18 years	Concomitant or isolated valvular surgery
Angiographically documented CAD	Aortic root reconstruction or Bentall procedure
Single-, double-, or triple-vessel coronary artery disease requiring surgical revascularization	Isolated congenital cardiac surgery
Primary surgical coronary revascularization	Redo cardiac surgery
Non-emergent surgery	Incomplete medical records or missing data for the primary study outcomes
Underwent either MICS-CABG or conventional OPCAB during the study period	—

Surgical techniques and cohort definitions

In the MICS-CABG group, surgical access was obtained through a 5-7 cm left anterolateral mini-thoracotomy at the fourth or fifth intercostal space. The left internal mammary artery (LIMA) was harvested using specialized retractors, and the coronary target vessels were exposed through the limited thoracotomy. All MICS-CABG procedures were performed using cardiopulmonary bypass (CPB) with femoral cannulation and aortic cross-clamping. CPB was used in all 40 patients to facilitate cardiac decompression, exposure, and multivessel coronary revascularization.

In the conventional OPCAB group, coronary revascularization was performed through a standard median sternotomy without the routine use of cardiopulmonary bypass. Coronary target vessels were exposed and stabilized using conventional epicardial stabilization techniques.

Data collection and outcome measures

Baseline variables extracted from the medical records included age, sex, body surface area (BSA), preoperative serum creatinine level, and pulmonary function parameters, including forced expiratory volume in one second (FEV1) and forced vital capacity (FVC).

Operative variables included the number of grafts performed and, where applicable, CPB and aortic cross-clamp times. Early postoperative outcomes included duration of mechanical ventilation, measured in hours, and postoperative chest tube drainage, measured in milliliters.

Safety outcomes included major adverse cardiac and cerebrovascular events (MACCE), re-exploration for postoperative bleeding, deep surgical site infection, acute respiratory failure, conversion from minimally invasive access to median sternotomy, and perioperative mortality. Statistical analysis was performed using IBM SPSS Statistics for Windows, version 26 (IBM Corp., Armonk, NY, USA). Continuous variables were assessed for distribution and are presented as mean ± standard deviation (SD). Between-group comparisons of continuous variables were performed using the independent-samples t-test for approximately normally distributed data; non-normally distributed variables were analyzed using the Mann-Whitney U test, as appropriate. Categorical variables are presented as frequencies and percentages and were compared using the chi-square test or Fisher's exact test when expected cell counts were small. Effect estimates were reported with 95% confidence intervals where appropriate. A two-sided p-value < 0.05 was considered statistically significant. Only patients with complete data for the variables included in the analysis were evaluated, and no imputation of missing data was performed. Given the retrospective design, small sample size, and systematic linkage between surgical approach and cardiopulmonary bypass strategy, no multivariable adjustment for confounding was performed. Accordingly, between-group findings were interpreted as unadjusted associations and not as evidence of an independent effect of surgical access.

## Results

Preoperative demographics and comorbidity profiles

A comprehensive review of the primary surgical registry yielded 80 fully documented patients matching the strict inclusion criteria for primary surgical revascularization (40 MICS-CABG cases and 40 conventional OPCAB. Valvular and congenital repair cases were systematically excluded due to distinct baseline physiology.

The mean cohort age was well distributed, highlighting a predominantly male presentation reflective of typical local CAD demographics. Preoperative systemic risk factors like type 2 diabetes mellitus and hypertension were highly prevalent across both cohorts. Baseline renal profiles remained optimized, with mean serum creatinine values hovering within normal biological ranges.

The comprehensive preoperative characteristics and distributed risk factors for both cohorts are outlined below in Table 2.

**Table 2:** Baseline Demographics and Preoperative Clinical Profiles MICS-CABG: minimally invasive coronary artery bypass grafting; OPCAB: off-pump coronary artery bypass grafting; LVEF: left ventricular ejection fraction; mg/dL: milligrams per deciliter.

Preoperative parameter	MICS-CABG (n = 40)	Conventional OPCAB (n = 40)	p-value
Age, years	55.1 ± 8.9	54.3 ± 12.8	0.747
Male sex, n (%)	36 (90.0)	31 (77.5)	0.225
Diabetes mellitus, n (%)	20 (50.0)	18 (45.0)	0.823
Hypertension, n (%)	20 (50.0)	22 (55.0)	0.823
Serum creatinine, mg/dL	1.03 ± 0.31	0.94 ± 0.35	0.227
Preoperative LVEF, %	53.0 ± 8.2	47.2 ± 11.2	0.01

Intraoperative and postoperative outcome analysis

Operative characteristics differed according to the surgical technique employed. In the MICS-CABG group, procedures were performed with cardiopulmonary bypass (CPB), with a mean CPB time of 182.0 ± 42.4 minutes and a mean aortic cross-clamp time of 104.9 ± 29.8 minutes. In contrast, patients in the conventional OPCAB group underwent revascularization without the use of CPB or aortic cross-clamping.

Differences were also observed in early postoperative recovery parameters. The mean duration of postoperative mechanical ventilation was 8.80 ± 3.97 hours in the MICS-CABG group compared with 4.22 ± 2.64 hours in the conventional OPCAB group. Mean chest tube drainage during the first 48 postoperative hours was 887.0 ± 611.8 mL in the MICS-CABG group and 328.9 ± 116.1 mL in the conventional OPCAB group. Table 3 compares intraoperative and postoperative Metrics. No perioperative mortality, stroke, deep surgical site infection, or conversion from minimally invasive access to median sternotomy was observed in either group.

**Table 3:** Comparative Intraoperative and Postoperative Metrics MICS-CABG: minimally invasive coronary artery bypass grafting; OPCAB: off-pump coronary artery bypass grafting; SD: standard deviation; CPB: cardiopulmonary bypass.

Parameter	MICS-CABG cohort (n = 40)	Conventional OPCAB cohort (n = 40)
Number of grafts, mean ± SD	2.8 ± 0.6	2.9 ± 0.9
Cardiopulmonary bypass use, n (%)	40 (100.0)	0 (0.0)
CPB time, minutes, mean ± SD	182.0 ± 42.4	Not applicable
Aortic cross-clamp time, minutes, mean ± SD	104.9 ± 29.8	Not applicable
Mechanical ventilation duration, hours, mean ± SD	8.80 ± 3.97	4.22 ± 2.64
Chest tube drainage within 48 hours, mL, mean ± SD	887.0 ± 611.8	328.9 ± 116.1
Major complications or perioperative mortality, n (%)	0 (0.0)	0 (0.0)

## Discussion

This single-center retrospective comparative cohort study describes our early institutional experience with MICS-CABG and compares perioperative outcomes with conventional OPCAB. A similar mean number of coronary grafts was achieved in the two groups (2.8 ± 0.6 vs 2.9 ± 0.9; p = 0.561). Postoperative mechanical ventilation was significantly longer in the MICS-CABG group than in the conventional OPCAB group (8.80 ± 3.97 vs 4.22 ± 2.64 hours; mean difference 4.58 hours, 95% CI 3.08-6.08; p < 0.001). Similarly, chest tube drainage during the first 48 postoperative hours was significantly greater in the MICS-CABG group (887.0 ± 611.8 vs 328.9 ± 116.1 mL; mean difference 558.1 mL, 95% CI 359.4-756.8; p < 0.001). However, these differences require cautious interpretation because all MICS-CABG procedures were performed using cardiopulmonary bypass (CPB) with aortic cross-clamping, whereas all conventional OPCAB procedures were performed off-pump. Surgical access and pump strategy were therefore systematically linked to treatment group, preventing their individual effects from being separated in this dataset. No perioperative mortality, stroke, deep surgical site infection, or conversion to median sternotomy was observed in either group. Collectively, these findings describe the feasibility and early perioperative profile of MICS-CABG at our institution rather than establishing superiority or inferiority relative to conventional OPCAB [1,2].

The feasibility of performing coronary revascularization through a limited thoracotomy has been demonstrated in previous studies. Early experiences established that multivessel coronary targets could be approached without median sternotomy, while subsequent series demonstrated refinement of coronary exposure, stabilization, conduit harvesting, and anastomotic techniques [2,3]. Ruel et al. reported successful multivessel MICS-CABG with angiographic assessment of grafts, providing evidence that minimally invasive access does not necessarily preclude extensive coronary revascularization [4]. Previous investigations have also emphasized the technical demands encountered during the introduction of MICS-CABG and the importance of accumulated procedural experience [5]. Subsequent institutional series have demonstrated that MICS-CABG programs can be introduced with acceptable early outcomes when accompanied by appropriate patient selection and structured operative strategies [6,7]. Long-term observational experience has further supported the potential durability of minimally invasive coronary revascularization in appropriately selected patients [8].

An important finding of the present study was the similarity in the mean number of grafts between the MICS-CABG and conventional OPCAB groups (2.8 ± 0.6 vs 2.9 ± 0.9; p = 0.561). Although the number of grafts alone cannot establish completeness or quality of myocardial revascularization, this finding indicates that a similar number of coronary targets was grafted in the two cohorts during the study period. This is relevant because limited access may raise concerns regarding exposure of the lateral and posterior coronary territories during multivessel MICS-CABG. Previous experience has demonstrated that multivessel grafting can be achieved through a left mini-thoracotomy when appropriate exposure, stabilization, conduit management, and operative strategies are employed [4,7,8]. Nevertheless, the present study did not include postoperative angiographic assessment of graft patency; therefore, similarity in graft number should not be interpreted as evidence of equivalent completeness or quality of revascularization.

Postoperative mechanical ventilation was significantly longer in the MICS-CABG group, with a mean between-group difference of 4.58 hours (95% CI 3.08-6.08; p < 0.001). This finding cannot, however, be attributed independently to minimally invasive surgical access. All MICS-CABG patients underwent CPB with aortic cross-clamping, whereas the conventional OPCAB group underwent revascularization without CPB. In addition, baseline LVEF was higher in the MICS-CABG group (53.0 ± 8.2% vs 47.2 ± 11.2%; p = 0.010), demonstrating that the groups were not completely comparable at baseline. Because treatment allocation was non-randomized and adjusted analyses could not be performed, the observed difference in ventilation duration may reflect the combined influence of CPB exposure, operative strategy, baseline differences, anesthetic management, case selection, and other measured or unmeasured factors. The result should therefore be regarded as an unadjusted between-group association rather than an independent effect of MICS-CABG.

The mean CPB and aortic cross-clamp times in the MICS-CABG cohort were 182.0 ± 42.4 minutes and 104.9 ± 29.8 minutes, respectively. These values describe operative performance during the early institutional implementation of the technique but should not be interpreted as evidence of a learning curve. Minimally invasive coronary surgery involves technical adaptations including operating through a restricted field, altered instrument trajectories, peripheral cannulation, and exposure of multiple coronary territories. Structured training, appropriate case selection, and coordinated development of the surgical, anesthetic, perfusion, and perioperative teams have been emphasized as important components of successful MICS-CABG implementation [9]. A systematic review and meta-analysis has also demonstrated measurable learning phases across minimally invasive cardiac procedures, although the number of cases required to achieve proficiency varies considerably according to procedure and institution [10]. The present study did not perform sequential case analysis, cumulative sum analysis, or another predefined learning-curve assessment; consequently, no conclusions regarding improvement with increasing procedural experience can be drawn from our aggregate operative times.

Chest tube drainage during the first 48 postoperative hours was also significantly greater in the MICS-CABG group, with a mean difference of 558.1 mL (95% CI 359.4-756.8; p < 0.001). As with ventilation duration, this finding should not be interpreted as an inherent consequence of minimally invasive access. Postoperative drainage may be influenced by several factors, including intercostal surgical access, internal mammary artery harvesting, systemic anticoagulation, CPB-related hemostatic effects, operative duration, and technical aspects of the procedure. Because CPB exposure was completely linked to the MICS-CABG group and no adjusted analysis was possible, the independent contribution of surgical access to postoperative drainage cannot be determined from these data. The substantial variability in drainage within the MICS-CABG group should also be considered when interpreting this finding.

The absence of conversion to median sternotomy among the 40 MICS-CABG patients is encouraging as a measure of technical feasibility during the early institutional experience. Conversion remains an important safety endpoint because sternotomy may become necessary in the setting of inadequate coronary exposure, uncontrolled bleeding, hemodynamic instability, or technical difficulty. Similarly, no perioperative mortality, stroke, or deep surgical site infection was observed in either cohort. These findings provide useful descriptive information regarding early procedural safety but should not be interpreted as demonstrating equivalence between MICS-CABG and conventional OPCAB. With only 40 patients in each group and no events for several major outcomes, the study has limited ability to evaluate uncommon complications or detect clinically important differences in these endpoints.

Avoidance of median sternotomy remains an important potential advantage of MICS-CABG. Long-term observational evidence suggests that minimally invasive coronary revascularization can provide favorable cardiovascular and functional outcomes in appropriately selected patients [11]. However, the clinical value of a minimally invasive approach cannot be assessed solely on the basis of incision size or early postoperative recovery. Effective surgical revascularization must also provide satisfactory graft quality and durability, survival, freedom from recurrent ischemia, and acceptable rates of subsequent cardiovascular events and repeat revascularization. Long-term studies of MICS-CABG have reported encouraging survival and cardiovascular outcomes [8,11].

The distinction between surgical access and operative strategy is particularly important when interpreting the present comparison. Evidence comparing on-pump and off-pump coronary revascularization demonstrates that pump strategy itself may influence perioperative and longer-term outcomes [12]. In our study, the MICS-CABG cohort uniformly underwent CPB and aortic cross-clamping, whereas the conventional comparator cohort uniformly underwent off-pump revascularization. Consequently, the two groups differ simultaneously in both surgical access and pump strategy. The present analysis therefore compares two operative strategies as they were practiced at our institution and does not isolate the effect of mini-thoracotomy versus median sternotomy.

Contemporary literature continues to define the role of MICS-CABG in coronary revascularization. A comprehensive review published in 2025 highlighted advances in patient selection, preoperative planning, conduit harvesting, coronary exposure, stabilization techniques, and perioperative management [13]. These developments are particularly relevant when establishing a new minimally invasive coronary program, where appropriate case selection and structured implementation may facilitate safe adoption. In this context, the present study provides experience from the early implementation of MICS-CABG in a developing healthcare setting rather than a formal assessment of procedural proficiency or a learning curve.

Prospective evidence is also emerging specifically for patients with multivessel CAD. A 2025 prospective study comparing 245 patients undergoing MICS-CABG with 158 patients undergoing OPCAB evaluated clinical and patient-reported outcomes after multivessel revascularization [14]. This is relevant to our cohort, in which the mean number of grafts in the MICS-CABG group was 2.8. Larger prospective comparative studies with appropriate adjustment for baseline and operative differences are needed to determine whether the potential benefits associated with avoiding sternotomy translate into meaningful clinical advantages while maintaining the completeness and durability expected from conventional surgical revascularization [14].

Long-term durability remains particularly important when evaluating MICS-CABG. Follow-up of a large MICS-CABG cohort demonstrated that repeat revascularization occurred in a minority of patients, with subsequent interventions related to both graft-related factors and progression of native CAD [15]. Evaluation of a developing MICS-CABG program should therefore extend beyond perioperative endpoints and include graft patency, recurrent angina, myocardial infarction, major adverse cardiac and cerebrovascular events, repeat revascularization, survival, functional recovery, quality of life, and return to normal activity [11,15]. These outcomes were not available in the present study and represent important targets for future prospective follow-up.

Our study provides early institutional experience from a developing healthcare setting, where implementation of minimally invasive coronary surgery may be influenced by access to specialized instrumentation, procedural volume, training opportunities, multidisciplinary expertise, and resource availability. Structured implementation, appropriate patient selection, proficiency in conventional coronary surgery, standardized operative protocols, mentorship, and coordinated team development may facilitate the safe introduction of these procedures [7,9,10,13]. The absence of perioperative mortality or emergency conversion in this selected cohort supports the technical feasibility of the approach at our institution; however, the present findings are insufficient to establish comparative effectiveness. Continued prospective evaluation with standardized outcome definitions and long-term follow-up is warranted.

Study limitations

This study has several important limitations. First, its retrospective, single-center design introduces the possibility of selection bias and limits generalizability. Treatment allocation was not randomized, and the clinical and anatomical characteristics influencing selection for MICS-CABG or conventional OPCAB may have resulted in systematic differences between the groups. Indeed, preoperative LVEF differed significantly between the cohorts. Because individual patient-level data were unavailable for adjusted re-analysis, multivariable regression, propensity-score methods, or other approaches to control for baseline confounding could not be performed. The reported comparisons should therefore be interpreted as unadjusted associations.

Second, the sample size was relatively small, with 40 patients in each group. Consequently, the study had limited ability to evaluate uncommon outcomes such as mortality, stroke, deep surgical site infection, re-exploration, or conversion to sternotomy. The absence of these events should therefore not be interpreted as demonstrating equivalent safety between the two operative strategies.

Third, the two cohorts differed fundamentally in operative strategy. All MICS-CABG procedures were performed using CPB with aortic cross-clamping, whereas all conventional OPCAB procedures were performed without CPB or aortic cross-clamping. Consequently, surgical access and pump strategy were systematically linked to treatment group, and their independent effects cannot be separated in this dataset. This represents a fundamental limitation of the comparative design and substantially restricts causal interpretation of the observed postoperative differences. In particular, the longer mechanical ventilation duration and greater chest tube drainage observed in the MICS-CABG group cannot be attributed independently to minimally invasive surgical access because these outcomes may also have been influenced by CPB exposure and other differences between the groups. The findings should therefore be interpreted as unadjusted comparisons between the two operative strategies as practiced at our institution rather than as estimates of the independent effect of minimally invasive versus sternotomy access.

Fourth, although the study represents the early institutional implementation of MICS-CABG, no formal learning-curve analysis was performed. The cases were not evaluated sequentially using cumulative sum analysis or another predefined methodology capable of determining changes in performance with increasing procedural experience. Therefore, the present study should not be interpreted as demonstrating a learning curve, and the aggregate CPB time, cross-clamp time, ventilation duration, and drainage values cannot establish improvement or deterioration with accumulated experience. Future studies should prospectively evaluate sequential procedural performance to characterize the institutional learning process.

Finally, long-term clinical and anatomical outcomes were unavailable. The study did not assess graft patency, recurrent angina, myocardial infarction, repeat revascularization, long-term major adverse cardiac and cerebrovascular events, survival, postoperative pain, quality of life, functional recovery, or return to normal activity. Future prospective studies should include larger patient populations, appropriate adjustment for baseline and operative confounders, sequential evaluation of institutional experience, and long-term assessment of graft patency using coronary computed tomography angiography or invasive coronary angiography.

## Conclusions

MICS-CABG was technically feasible for multivessel coronary revascularization during the early institutional experience at our center, with no perioperative mortality, stroke, deep surgical site infection, or conversion to median sternotomy observed in the MICS-CABG cohort. However, MICS-CABG was associated with longer postoperative mechanical ventilation and greater chest tube drainage compared with conventional OPCAB. These findings should be interpreted in the context of the institutional learning phase and the use of cardiopulmonary bypass in the MICS-CABG group. Larger prospective studies with long-term follow-up are required to evaluate graft patency, clinical outcomes, and the durability of this minimally invasive approach.
